# Health Behaviors and Mental Health during the COVID-19 Pandemic: Evidence from the English Longitudinal Study of Aging

**DOI:** 10.1177/07334648231159373

**Published:** 2023-02-28

**Authors:** Giorgio Di Gessa, Paola Zaninotto

**Affiliations:** 1 Institute of Epidemiology and Health Care, Department of Epidemiology and Public Health, 4919 University College London, London, UK

**Keywords:** ELSA, older people, physical activity, sleeping, eating, depression, life satisfaction, quality of life

## Abstract

Stay-at-home and lockdown measures during the COVID-19 pandemic had an impact on health-related behaviors which in turn posed a risk to mental health, particularly among older people. In this study, we investigated how changes to health behaviors (physical activity, sleeping, eating, and drinking) impacted mental health (depression, quality of life, and life satisfaction) during and beyond the initial phase of the COVID-19 lockdown. Using data from Wave 9 (2018/19) and two COVID-19 sub-studies (June/July 2020; November/December 2020) of the English Longitudinal Study of Ageing (*N* = 4989), we found that health behaviors changes during lockdown were associated with poorer mental health in June/July 2020. However, in November/December 2020, higher depression, lower quality of life, and lower life satisfaction were more likely only among respondents who reported less physical activity, eating more, changes in sleep patterns, and drinking more alcohol. Public health programs should support healthy behaviors as we emerge from the pandemic.

**What this paper adds**

- A considerable percentage of older people in England were engaged in more unhealthy behaviors during the 2020 lockdown.
- Those who reported changes (in either direction) to their health behaviors were more likely to report higher odds of depression, lower quality of life, and lower life satisfaction in June/July 2020.
- These negative impacts were also found in the longer period (November/December 2020), suggesting that healthy behaviors foster mental wellbeing over time.

**Applications of study findings**

- This research gives insights into the importance of health behaviors among older people, and how sudden changes in health behaviors can have negative impacts on mental health.
- Incorporating a longitudinal approach to the study of the complex interplay between health behaviors and mental health helps to provide a better understanding of short and longer-term effects and to account for confounding characteristics.
- Developing and strengthening public health programs that encourage engagement in health-promoting behaviors among older people and the general population is crucial to reduce the COVID-19 crisis–related unintended long-term side effects on mental health.

## Introduction

Soon after the COVID-19 outbreak, governments introduced substantial restrictions on people’s movements, such as working from home, school closures, and limitations to see friends and family to protect people from infection and reduce the spread of the virus in the community (Hale et al., 2021). As risks of serious illness and death deriving from COVID-19 increased with age and pre-existing diseases (also strongly correlated with age) (Jordan et al., 2020), although these general guidelines applied to all, there were clear recommendations that particularly older people should stay indoors, limit their travels and movements, as well as limit physical interactions with others (Ayalon, 2020; Perra, 2021). When the United Kingdom (UK) government announced the first lockdown on the 23rd of March 2020, all those over 70 and those deemed “clinically vulnerable” were advised to stay indoors and limit their interactions with others for 12 weeks, and 2.2 million clinically extremely vulnerable people (two thirds of which aged 60 and older) were ordered to shield until at least the end of June 2020. Policies restricting social contact and human interaction have clearly posed a risk to mental health and wellbeing, and research has shown deteriorating mental health, especially among those with pre-existing mental health conditions, low social support, and at-risk groups (Araki, 2022; Armitage & Nellums, 2020; Brooks et al., 2020, 2022; Daly et al., 2022; Di Gessa & Price, 2021, 2022; Patel et al., 2022; Pfefferbaum & North, 2020; Pierce et al., 2020; Polenick et al., 2021; Steptoe & Di Gessa, 2021).

Government measures also had an impact on health-related behaviors and daily routines, such as alcohol consumption and physical exercise. Within 10 days of the World Health Organization’s (WHO) declaration of a pandemic in 2020, the mean value of daily steps decreased by about 6%, and within 30 days by about 27% (Tison et al., 2020). Although evidence suggests changes in either direction, early cross-sectional data mostly showed reductions in leisure-time sports activity, as well as more time was spent on sedentary activities and on screen (Choi et al., 2023; Colley et al., 2020; Gallè et al., 2021; Mutz & Gerke, 2021; Qin et al., 2020; Stockwell et al., 2021). The COVID-19 lockdown has also impacted eating habits (Bennett et al., 2021; González-Monroy et al., 2021): for instance, a web-based survey conducted in April 2020 in Italy found that a third of respondents had more appetite, with roughly an equal percentage of respondents declaring to eat more (37%) or less (36%) healthy food such as fruit, vegetables, and legumes (Di Renzo et al., 2020). Several studies on individual-level changes in alcohol use have also shown that although most people did not change their drinking amount, among those who did, a larger proportion increased consumption and drinking frequency (Pollard et al., 2020; Sallie et al., 2020; Tran et al., 2020). A systematic review of longitudinal studies that compared changes in eating behavior and drinking before and after the COVID-19 pandemic outbreak found a more frequent intake of food and alcohol consumption (González-Monroy et al., 2021). The pandemic has also had an impact on sleep quality, timing, and duration, with early studies showing an overall reduction in night-time sleep as well as a deterioration of sleep quality (Gupta et al., 2020; Jahrami et al., 2021; Petrov et al., 2021).

The relationship between health behaviors and mental health is well established in the literature. Although health behaviors are strongly intertwined with mental health in a bi-directional way, growing evidence suggests that key health behaviors such as being physically active, reduced alcohol consumption, or healthy diet are associated with lower prevalence of detrimental mental health outcomes as well as reduced risk of depression (Arora & Grey, 2020; Boden & Fergusson, 2011; Harrington et al., 2009; Mammen & Faulkner, 2013; Velten et al., 2014). Since the COVID-19 pandemic, several studies have shown that changes in health behaviors were associated with poorer psychological health and wellbeing, particularly reduction of total physical activity (Maugeri et al., 2020), sleep problems including poorer sleep quality, sleep disruptions, and changes in sleep duration (Alimoradi et al., 2021; Coiro et al., 2021), changes in individual’s eating behaviors and switching to less-healthy eating habits and comfort eating (Chan & Chiu, 2022; Ingram et al., 2020), as well as increased alcohol consumption (Jacob et al., 2021).

However, important lacunae remain in our understanding of the associations between changes in behaviors and mental health. First, most of the studies so far are cross-sectional and have used convenience or small-scale samples. Second, because of the nature of these studies, many could not account for pre-pandemic mental and physical characteristics which could potentially lead to a higher risk of changing health behaviors during the pandemic as well as to deteriorating mental health and wellbeing. Third, the effects of health behavior changes on mental health were mostly studied during the initial phase of the pandemic, with few studies that have examined the longer-term effect. Finally, although a few studies have provided information on several age groups, few studies have focused on older adults, despite this group accounting for about 20% of the UK population and having been particularly encouraged to adopt precautionary behaviors such as physical distancing and staying at home during all phases of the pandemic to limit admissions to Intensive Care Units and deaths from COVID-19–related conditions.

Producing a better understanding of how changes in (modifiable) health behaviors among older people relate to mental health is key to developing appropriate and targeted policy responses as we aim to build back society and restore the wellbeing of our populations. Therefore, in this paper, we aim to understand how behavioral changes related to poorer mental health, both in the short and longer run of the COVID-19 pandemic, analyzing nationally representative longitudinal data, and controlling for pre-pandemic health.

## Methods

### Study Population

We used the most recent pre-pandemic data (wave 9, collected in 2018/19) and the two waves of the COVID-19 sub-study (collected in June/July and Nov/Dec 2020 respectively) of the English Longitudinal Study of Ageing (ELSA) (Banks et al., 2019). English Longitudinal Study of Ageing is a longitudinal biennial survey representative of individuals aged 50 and over in private households. During the pandemic, ELSA members were invited to participate online or by CATI (Computer-Assisted Telephone Interviewing) to the COVID-19 sub-study (75% response rate in both waves, 94% longitudinal response rate). Analyses were based on core members who participated in both COVID-19 waves with available information also in wave 9 (*N* = 4989). Further details of the survey’s sampling frame and methodology can be found at www.elsa-project.ac.uk. English Longitudinal Study of Ageing was approved by the London Multicenter Research Ethics Committee (MREC/01/2/91), with information on the ethical approval received for each wave of ELSA available at https://www.elsa-project.ac.uk/ethical-approval. Informed consent was obtained from all participants. All data are available through the UK Data Service (SN 8688 and 5050).

### Main Measurements of Interest

#### Changes in health behaviors

Respondents were asked in June/July 2020 (baseline) whether some of their health behaviors changed since the COVID-19 outbreak began in February 2020. In particular, they were asked to report if they had been doing physical exercise, eating, and sleeping “less than usual,” “about the same,” or “more than usual.” Comparable questions were also asked about drinking, though changes in behaviors for this variable were only collected among those who were drinking alcohol at the time of the interview.

#### Mental health

We considered three outcome measures of mental health assessed at both COVID-19 sub-studies: depressive symptoms, quality of life, and life satisfaction. Symptoms of depression were measured by an abbreviated version of the validated Center for Epidemiologic Studies Depression (CES-D) Scale (Beekman et al., 1997). The CES-D scale is not a diagnostic instrument for clinical depression but can be used to identify people “at risk” of depression in population-based studies (Radloff, 1977). This short version has good internal consistency (Cronbach’s α > 0.95) and comparable psychometric properties to the full 20-item CES-D (Karim et al., 2015). The scale includes eight binary (no/yes) questions that enquire about whether respondents experienced any depressive symptoms, such as feeling sad or having restless sleep, in the week prior to interview. We classified respondents who reported four or more depressive symptoms on the CES-D scale as with elevated depressive symptoms (Turvey et al., 1999; Zivin et al., 2010). Subjective quality of life (QoL) was evaluated using the 12-item Control, Autonomy, Self-realization and Pleasure (CASP-12) scale. This is an abbreviated measure of the validated CASP-19 scale which was specifically designed for individuals in later life and used in a wide variety of aging surveys (Hyde et al., 2003). CASP-12 contains 12 Likert-scaled questions measuring older people’s control and autonomy as well as self-realization through pleasurable activities. The possible range of CASP-12 scores is from 0 to 36, with higher scores indicating greater wellbeing; CASP-12 is treated as a continuous variable. Finally, we considered life satisfaction as a measure of personal wellbeing assessed using the Office for National Statistics (ONS) wellbeing scale (“On a scale of 0–10, where 0 is ‘not at all’ and 10 is ‘very’, how satisfied are you with your life nowadays?”). This allows respondents to integrate and weigh various life domains the way they choose (Pavot & Diener, 1993).

#### Covariates

Our analyses controlled for a wide range of demographic, socio-economic characteristics, health, and social support characteristics. These included age; sex; and ethnicity (White vs. non-White participants due to data constraints in ELSA). Respondents’ socio-economic characteristics were captured by education, pre-pandemic wealth, living arrangements, and employment status during the pandemic. Educational level was recoded into low (below university) and high (university or above) following the International Standard Classification of Education (http://www.uis.unesco.org/). We categorized respondents by quintiles of wealth (total net non-pension non-housing wealth). Living arrangements distinguished respondents by whether they were living with their partner or not. Employment distinguished retired, in paid work, furloughed, and other (including homemakers, unemployed, and sick or disabled). Moreover, we considered a binary variable indicating whether respondents (and their family members) had always enough of the kinds of foods they wanted to eat during the pandemic or not. We also considered as measure of social support whether ELSA respondents reported real-time contact (by telephone or video calling) with family and friends at least weekly or less than once a week or never in the month prior to the interview. Finally, we controlled for whether respondents had negative experiences of COVID-19 (proxied by whether respondents or any of their friends or relatives had been hospitalized or a friend or relative had died because of COVID-19).

We also accounted for pre-pandemic health. In particular, we controlled for disability (having impairments with basic or instrumental activities of daily living) and clinical vulnerability to COVID-19 (defined as reporting chronic conditions such as lung disease, asthma, coronary heart disease, Parkinson’s disease, multiple sclerosis, diabetes; weakened immune system as a result of cancer treatment in the previous 2 years; BMI of 40 or above; or having been advised to shield by their GP/NHS) (Di Gessa & Price, 2021; UK GOV, 2020). We further controlled for pre-pandemic measures of mental health (see above for derivation).

### Statistical Analyses

Following descriptive analysis, we investigated the associations between changes in health behaviors and mental health, both cross-sectionally by analyzing outcomes measured in the first COVID-19 sub-study assessment (June/July 2020), and longitudinally using measures collected in Nov/Dec 2020. We used logistic models for depression and linear regression models for CASP-12 and life satisfaction. We considered each change in health behavior separately, and all models adjusted for demographic and socio-economic characteristics, health, and social support, as well as for pre-pandemic relevant measures of mental health. We performed all analyses using Stata 16. Cross-sectional and longitudinal sampling weights were employed to account for different probabilities of being included in the sample and for nonresponse to the survey.

## Results

### Descriptive Statistics

Table 1 describes the sociodemographic and health characteristics of our analytical sample (*N* = 4989), as well as how their health behaviors changed during the pandemic. Most participants did not report any changes in their lifestyle behaviors during the first months of the COVID-19 pandemic. However, 35% reported engaging less in physical activity with almost one in five reporting more physical activity. Nineteen per cent of respondents reported eating more whereas less than one in ten had been eating less than usual. Almost 23% reported less sleep and roughly an equal percentage of ELSA respondents reported drinking either more or less than usual since the coronavirus outbreak.

**Table 1.** Sample Characteristics.

**Changes in health behaviours in COVID-19—Jun/Jul 2020**	% (*N*)
Physical activity – Less	35.2 (1764)
– Same	46.4 (2287)
– More	18.4 (931)
Eating – Less	9.4 (453)
– Same	71.6 (3693)
– More	19.0 (842)
Sleeping – Less	22.7 (1009)
– Same	68.2 (3572)
– More	9.1 (407)
Not drinking alcohol	37.0 (1751)
Drinking – Less	14.4 (701)
– Same	35.6 (19.3)
– More	13.0 (610)
**Sociodemographic and economic characteristics**	
50–59	29.9 (755)
60–69	31.1 (1565)
70–79	25.3 (1890)
80 and older	13.7 (779)
Female	53.0 (2851)
Non-White	6.1 (176)
High educational qualification	21.7 (1279)
Middle educational qualification	49.9 (2509)
Low educational qualification	28.4 (1201)
Highest wealth quintile	20.7 (1233)
2^nd^ wealth quintile	21.2 (1217)
3^rd^ wealth quintile	20.3 (1075)
4^th^ wealth quintile	19.2 (835)
Lowest wealth quintile	18.6 (629)
Retired	52.8 (3449)
Employed	28.4 (900)
Furloughed	8.4 (300)
Sick/Disabled/Unemployed/Other	10.4 (339)
Not always with enough food of the kind wanted	15.3 (689)
Living with partner	65.8 (3326)
Infrequent phone/videocall contacts with friends and family	7.4 (340)
Negative experience of COVID-19	8.3 (382)
**Pre-pandemic health characteristics (at Wave 9)**	
4 or more CES-D depressive symptoms	12.2 (511)
Mean CASP-12 (SD)	26.33 (6.27)
Mean life satisfaction (SD)	7.43 (2.22)
Clinically vulnerable to COVID-19	38.1 (1887)
Disabled	21.6 (1028)
**Total number of respondents (*N*)**	**4989**

*Notes:* CASP-12 = 12-item Control, Autonomy, Self-realization and Pleasure; CES-D = Center for Epidemiological Studies Depression; COVID-19 = coronavirus disease 2019; ELSA = English Longitudinal Study of Ageing; SD = standard deviation. Source: ELSA, COVID-19 Sub-study Wave 1 (June/July 2020) and Wave 9 (2018–2019). Weighted data.

Table 2 shows that mental health measured in the first COVID-19 ELSA sub-study revealed substantial variation by changes in health behaviors. Overall, respondents who reported about the same behaviors since the coronavirus outbreak in February 2020 also had the lowest percentages of elevated depressive symptoms, and the highest quality of life and life satisfaction. For instance, respondents eating about the same reported the best mental health (with 17.4% having elevated depressive symptoms, a mean CASP-12 quality of life of 26.2 and a mean life satisfaction of 7.2), whereas those who ate less or more reported poorer mental health (with 37.5% of those eating less reporting four or more depressive symptoms and a mean CASP-12 score of 23). The only exception to this pattern is with physical activity: for this behavior, it is older people who reported more exercise who had the best mental health and wellbeing (see Table 2 for full details). As expected, we observed similar variations also when we considered pre-pandemic mental health, with respondents whose health behaviors remained stable during the first months of the pandemic more likely to also have had better pre-pandemic mental health (see Supplementary Table S1 for full details).

**Table 2.** Unadjusted Mental Health by Changes in Health Behaviors During the First Months of the COVID-19 Pandemic.

	**% Elevated CES-D symptoms**	**Mean quality of life (CASP-12)**	**Mean life satisfaction**
Physical activity – Less	27.8	24.30	6.64
– Same	19.5	25.79	7.23
– More	14.2	27.49	7.28
Eating – Less	37.5	23.02	6.46
– Same	17.4	26.17	7.20
– More	29.4	24.45	6.64
Sleeping – Less	42.8	22.37	6.02
– Same	14.1	26.75	7.40
– More	24.7	24.42	6.78
Not drinking alcohol	28.9	24.03	6.82
Drinking – Less	19.8	25.66	6.91
– Same	14.8	26.84	7.35
– More	21.3	26.15	6.85
*Overall*	**21.6**	**25.54**	**7.03**

*Notes:* CASP-12 = 12-item Control, Autonomy, Self-realization and Pleasure; CES-D = Center for Epidemiological Studies Depression; COVID-19 = coronavirus disease 2019; ELSA = English Longitudinal Study of Ageing. Source: ELSA COVID-19 sub-study Wave 1 (June/July 2020). Weighted data.

### Multivariable Analyses

**Figure 1.**
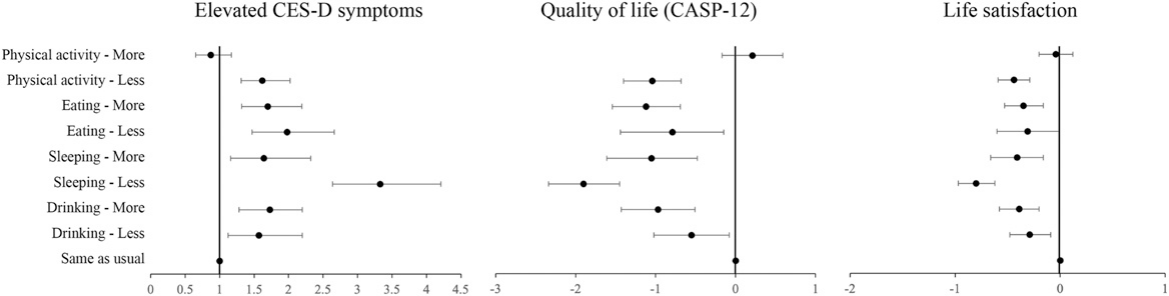
Cross-sectional associations between changes in health behaviors and mental health in June/July 2020. *Source*: ELSA, COVID-19 Substudy Wave 1 (June/July 2020) and Wave 9 (2018-2019). Detailed models can be found in the Supplementary Tables S2-S5.

To investigate how changes in health behaviors during the pandemic were associated with mental health, we used multivariable logistic and linear regressions. Cross-sectional results are presented for the main variables of interest in Figure 1 (with full results in Supplementary Tables S2-S5). Analyses show that, even after accounting for sociodemographic and economic characteristics as well as pre-pandemic physical and mental health, changes in health behaviors were associated with detrimental mental health in June/July 2020. Compared to respondents who did not change their habits, respondents who reported a change in either direction (i.e., who reported doing either *more* or *less* than before) of their sleeping, eating, and drinking behaviors were more likely to report poorer mental health outcomes. For instance, compared to those who reported no change, older people who since the coronavirus outbreak slept less or more than usual were more likely to report elevated depressive symptoms (OR = 3.33, 95%CI = 2.64 to 4.21 for less, and OR = 1.64, 95%CI = 1.16 to 2.32 for more), lower quality of life (B = −1.90, 95%CI = −2.34 to −1.45 for less, and B = −1.05, 95%CI = −1.61 to −0.48 for more), and lower life satisfaction (B = −0.80, 95%CI = −0.97 to −0.62 for less, and B = −0.41, 95%CI = −0.66 to −0.16 for more). For physical activity, we found that, compared to those doing about the same physical activity, only respondents reporting less than usual had higher odds of depression (OR = 1.62, 95%CI = 1.31–2.02), lower average quality of life (B = −1.04, 95%CI: −1.40 to −0.68), and lower life satisfaction (B = −0.44, 95%CI = −0.59 to −0.29).

**Figure 2.**
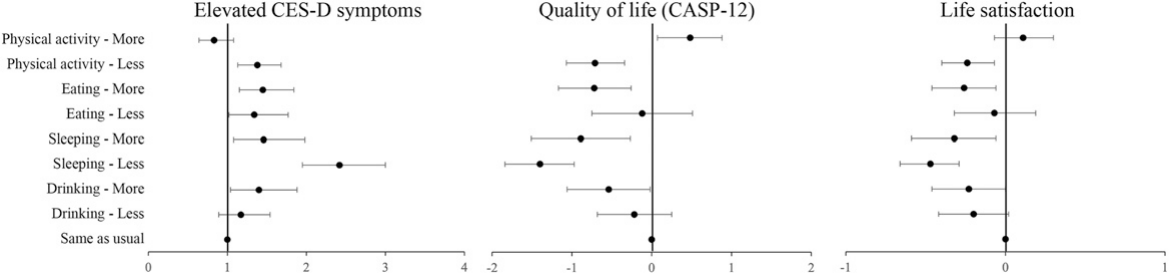
Longitudinal associations between changes in health behaviors in June/July 2020 and mental health in November/December 2020. *Source*: ELSA, COVID-19 Substudy Waves 2 (Nov/Dec 2020) and 1 (June/July 2020), and Wave 9 (2018-2019). Detailed models can be found in the Supplementary Tables S6-S9.

Longitudinal associations between changes in health behaviors and mental health measured in November/December 2020 are shown in Figure 2 (with full results in Supplementary Tables S6-S9). Doing less physical activity, eating more, changes in sleep, and drinking more than usual in the first months of the pandemic were negatively associated with all three measures of mental health also longitudinally, even after accounting for demographic, socio-economic, and health characteristics. Moreover, results suggest that compared to those who reported no change, respondents eating less than usual had increased odds of elevated depressive symptoms (OR = 1.34, 95%CI = 1.02–1.77), whereas those doing more physical activity had higher quality of life (B = 0.48, 95CI% = 0.07–0.88).

## Discussion

In response to the COVID-19 pandemic, governments adopted several strategies to encourage people to stay at home and limit their physical interactions. These measures had a considerable impact on several aspects of daily human life, including lifestyles and healthy behaviors that are known to have a positive impact on mental health and wellbeing. In this paper, therefore, we investigate whether changes in health behaviors soon after the beginning of the pandemic are associated with three measures of mental health (namely, depression, quality of life, and life satisfaction) among older people in England during the first 8/9 months of the pandemic.

Our analyses show that a considerable percentage of older people in England were engaged in more unhealthy behaviors during the lockdown, such as doing less physical activity than usual, eating more, drinking more alcohol, and sleeping both less and more than usual. Overall, compared to those who maintained unchanged their behaviors, those who reported changes in either direction to their health behaviors (except those doing more physical exercise) were more likely to report higher odds of depression, lower quality of life, and lower life satisfaction in June/July 2020. Moreover, we found that doing less physical activity, eating more, changes in sleep, and drinking more than usual were also associated longitudinally with poorer mental health assessed in November/December 2020. Our findings are in line with studies that have assessed the range of downstream psychosocial consequences of the (largely inevitable) modifications to health behaviors soon after the COVID-19 outbreak and its associated containment measures (Alimoradi et al., 2021; Arora & Grey, 2020; Chan & Chiu, 2022; Coiro et al., 2021; Ingram et al., 2020; Jacob et al., 2021; Maugeri et al., 2020), and confirm the importance of health behaviors for mental health. Moreover, our study provides an insight not just into a cross-sectional assessment of the associations between changes to health behavior and mental health, but also an understanding of the long(er)-term consequences of such changes. The findings that changes in health behaviors are associated with detrimental mental health also in the longer period are consistent with the literature suggesting that healthy behaviors can foster mental wellbeing over time

## Strength and Limitations

This study has several strengths, including the use of a nationally representative study of older people living in private households in England; the investigation of the immediate and long-term associations between changes to several measures of health behaviors and mental health; and the inclusion of pre-pandemic measures of health and mental health which was not controlled for in most COVID-19 studies. However, there are several limitations to consider. Changes in healthy behaviors were ascertained using a one-item measure so analyses are limited in detecting nuanced changes. Moreover, the phrasing of the questions compared changes to the individuals’ “usual” behaviors, meaning that we could only study self-perceived changes rather than objective changes between before and during the pandemic. In addition, ELSA did not collect information about respondents’ health behaviors during the pandemic, so it is not possible to understand the level and frequency of the various behaviors considered in this study and how they relate to changes. For instance, in pre-pandemic times, in England, the percentage of adults aged 65–74 meeting the recommended physical activity levels was about 55%, and less than a third of those aged 65 and older were eating the recommended five portions of fruit and vegetables per day (https://digital.nhs.uk/data). Similarly, no questions were asked about why behaviors changed: it is plausible that the reasons behind those changes (such as loss of appetite, stress, and fear to leave the house to exercise) may further affect their relationships with mental health, or at least provide some explanations for the associations found. Furthermore, in this study, we considered each change in behavior separately. However, it is important to recognize that changes in one domain are likely to result in collateral changes in others and future studies should consider composite measures of changes to health behaviors. Furthermore, in our study, we considered changes in the first few months of the pandemic, and we could not assess whether with the easing of the restrictions health behaviors improved and whether these, in turn, resulted in improved mental health effects. Finally, although in our study we controlled for respondents’ socio-economic characteristics, future studies should also consider whether and to what extent changes in both health behaviors and mental health during the pandemic were socially patterned to better develop targeted strategies that could help reduce the impact of the COVID-19 outbreak.

In conclusion, our study shows that changes in health behaviors were associated with poorer mental health, both in the short and the longer term. As we emerge from the current pandemic, public health programs should encourage engagement in health-promoting behaviors among older people and the general population, as these might help reduce the COVID-19 crisis–related unintended long-term side effects on mental health.

## Supplemental Material

Supplemental Material - Health Behaviors and Mental Health during the COVID-19 Pandemic: Evidence from the English Longitudinal Study of Aging Click here for additional data file. Supplemental Material for Health Behaviors and Mental Health during the COVID-19 Pandemic: Evidence from the English Longitudinal Study of Aging by Giorgio Di Gessa and Paola Zaninotto in Journal of Applied Gerontology
