# Supplementary material for: Health Behaviors and Mental Health during the COVID-19 Pandemic: Evidence from the English Longitudinal Study of Aging
Source: J Appl Gerontol. 2023 Feb 28;42(7):1541–50. doi: 10.1177/07334648231159373 (PMC9978235; doi:10.1177/07334648231159373)
Supplement: Supplemental Material - Health Behaviors and Mental Health during the COVID-19 Pandemic: Evidence from the English Longitudinal Study of Aging [file sj-pdf-1-jag-10.1177_07334648231159373.pdf]

Supplementary Table S1. Unadjusted pre-pandemic mental health by changes in health behaviours during the first months of the COVID-19 pandemic

	% Elevated depressive symptoms (CES-D)	Mean quality of life (CASP-12)	Mean life satisfaction
Physical activity – Less	12.9	25.69	7.23
– Same	13.1	26.14	7.43
– More	8.3	28.04	7.80
Eating – Less	22.8	24.01	6.74
– Same	10.2	26.68	7.56
– More	14.5	26.14	7.27
Sleeping – Less	22.5	24.04	6.66
– Same	8.2	27.16	7.68
– More	16.6	25.67	7.35
Not drinking alcohol	16.9	24.72	7.03
Drinking – Less	10.2	26.38	7.36
– Same	9.4	27.46	7.80
– More	9.0	27.67	7.59
	**12.1**	**26.33**	**7.43**

Source: ELSA COVID-19 sub-study Wave 1 and Wave 9 (pre-pandemic mental health measures). Weighted data

Supplementary Table S2. Cross-sectional associations between changes in physical activity and mental health. Fully-adjusted logistic and linear regression models

	**Elevated CES-D symptoms**	**Quality of life (CASP-12)**	**Life satisfaction**
Physical activity – Less	1.62*** [1.31,2.02]	-1.04*** [-1.40,-0.68]	-0.44*** [-0.59,-0.29]
Physical activity – Same	*Ref*	*Ref*	*Ref*
Physical activity – More	0.87 [0.65,1.17]	0.21 [-0.17,0.59]	-0.04 [-0.20,0.12]
Female	1.60*** [1.28,2.00]	-0.42* [-0.75,-0.09]	-0.26*** [-0.39,-0.12]
Age	0.97*** [0.96,0.99]	0.01 [-0.02,0.03]	0.02*** [0.01,0.03]
Non-White	0.64 [0.35,1.15]	0.81 [-0.12,1.75]	0.55** [0.20,0.91]
Medium education (Ref: High)	1.13 [0.86,1.49]	0.25 [-0.12,0.61]	0.27** [0.10,0.43]
Low education	1.01 [0.73,1.38]	0.61* [0.14,1.09]	0.43*** [0.23,0.64]
2nd lowest quintile (Ref: lowest)	0.76 [0.53,1.10]	0.54 [-0.08,1.16]	0.14 [-0.12,0.39]
3rd wealth quintile	0.91 [0.65,1.28]	0.87** [0.24,1.49]	0.05 [-0.19,0.30]
4th wealth quintile	0.76 [0.54,1.07]	0.18 [-0.43,0.79]	-0.14 [-0.39,0.11]
Highest wealth quintile	0.83 [0.59,1.19]	0.53 [-0.08,1.14]	-0.18 [-0.43,0.06]
Employed (Ref: Retired)	0.64** [0.46,0.90]	0.35 [-0.13,0.83]	0.32** [0.12,0.52]
Furloughed	0.62* [0.39,0.99]	-0.21 [-0.86,0.44]	0.04 [-0.26,0.34]
Other employment	1.70** [1.14,2.53]	-1.44*** [-2.20,-0.68]	-0.44** [-0.76,-0.12]
Always with enough food	0.60*** [0.46,0.78]	1.83*** [1.35,2.32]	0.39*** [0.17,0.60]
Living with partner	0.63*** [0.51,0.78]	-0.07 [-0.42,0.29]	0.15 [-0.00,0.30]
Infrequent contacts	1.20 [0.82,1.76]	0.01 [-0.71,0.72]	0.08 [-0.20,0.35]
Negative experiences of COVID-19	1.27 [0.86,1.87]	-0.30 [-0.90,0.30]	-0.21 [-0.50,0.08]
Clinically vulnerable to COVID-19	1.20 [0.97,1.49]	-0.32 [-0.66,0.02]	-0.00 [-0.15,0.14]
Disability	1.88*** [1.46,2.42]	-1.17*** [-1.65,-0.69]	-0.07 [-0.27,0.12]
Pre-pandemic relevant mental health	5.22*** [3.93,6.94]	0.64*** [0.60,0.67]	0.43*** [0.39,0.47]
Constant		7.64*** [6.40,8.88]	3.52*** [3.08,3.96]
Observations	*4,957*	*4,701*	*4,647*

Sources: ELSA, COVID-19 sub-study Wave 1 (June/July 2020) and Wave 9 (2018/19).

Notes: Odds Ratios [and 95% confidence intervals (CIs)] reported for elevated depressive symptoms, and Beta coefficients [and 95% CIs] for the continuous outcome variables 'Quality of life' and 'Life Satisfaction'. For both continous outcomes, the relevant health questions in Wave 9 were asked in the self-completion questionnaire (hence, the smaller sample size). * $p < 0.05$, ** $p < 0.01$, *** $p < 0.001$. Weighted data.

Supplementary Table S3. Cross-sectional associations between changes in eating and mental health. Fully-adjusted logistic and linear regression models

	**Elevated CES-D symptoms**	**Quality of life (CASP-12)**	**Life satisfaction**
Eating – Less	1.98*** [1.47,2.66]	-0.79* [-1.44,-0.15]	-0.31* [-0.60,-0.01]
Eating – Same	*Ref*	*Ref*	*Ref*
Eating – More	1.70*** [1.32,2.19]	-1.12*** [-1.54,-0.69]	-0.35*** [-0.53,-0.16]
Female	1.57*** [1.26,1.96]	-0.40* [-0.73,-0.08]	-0.26*** [-0.39,-0.12]
Age	0.98*** [0.96,0.99]	-0.00 [-0.02,0.02]	0.01** [0.00,0.02]
Non-White	0.68 [0.38,1.21]	0.77 [-0.14,1.69]	0.55** [0.20,0.89]
Medium education (Ref: High)	1.14 [0.87,1.49]	0.24 [-0.13,0.60]	0.27** [0.10,0.43]
Low education	1.01 [0.74,1.39]	0.60* [0.13,1.07]	0.44*** [0.23,0.64]
2nd lowest quintile (Ref: lowest)	0.78 [0.54,1.12]	0.54 [-0.08,1.16]	0.14 [-0.11,0.39]
3rd wealth quintile	0.92 [0.66,1.28]	0.90** [0.28,1.52]	0.07 [-0.18,0.31]
4th wealth quintile	0.76 [0.54,1.07]	0.22 [-0.40,0.83]	-0.12 [-0.37,0.13]
Highest wealth quintile	0.83 [0.58,1.18]	0.58 [-0.03,1.18]	-0.16 [-0.41,0.08]
Employed (Ref: Retired)	0.63** [0.45,0.88]	0.36 [-0.12,0.84]	0.33** [0.13,0.53]
Furloughed	0.62* [0.39,0.98]	-0.19 [-0.83,0.46]	0.04 [-0.26,0.34]
Other employment	1.72** [1.16,2.56]	-1.40*** [-2.15,-0.64]	-0.42* [-0.74,-0.10]
Always with enough food	0.61*** [0.47,0.80]	1.83*** [1.34,2.32]	0.39*** [0.17,0.60]
Living with partner	0.64*** [0.52,0.80]	-0.10 [-0.45,0.26]	0.14 [-0.01,0.29]
Infrequent contacts	1.28 [0.87,1.87]	-0.15 [-0.87,0.58]	0.02 [-0.26,0.30]
Negative experiences of COVID-19	1.23 [0.83,1.80]	-0.26 [-0.87,0.34]	-0.20 [-0.49,0.09]
Clinically vulnerable to COVID-19	1.21 [0.98,1.49]	-0.37* [-0.70,-0.03]	-0.02 [-0.16,0.13]
Disability	1.92*** [1.50,2.47]	-1.20*** [-1.68,-0.71]	-0.07 [-0.27,0.12]
Pre-pandemic relevant mental health	4.92*** [3.71,6.54]	0.64*** [0.60,0.67]	0.43*** [0.39,0.47]
Constant		7.56*** [6.34,8.78]	3.44*** [2.99,3.88]
Observations	*4,962*	*4,705*	*4,648*

Sources: ELSA, COVID-19 sub-study Wave 1 (June/July 2020) and Wave 9 (2018/19).
Notes: Odds Ratios [and 95% confidence intervals (CIs)] reported for elevated depressive symptoms, and Beta coefficients [and 95% CIs] for the continuous outcome variables 'Quality of life' and 'Life Satisfaction'. For both continous outcomes, the relevant health questions in Wave 9 were asked in the self-completion questionnaire (hence, the smaller sample size). * $p < 0.05$, ** $p < 0.01$, *** $p < 0.001$. Weighted data.

Supplementary Table S4. Cross-sectional associations between changes in sleeping and mental health. Fully-adjusted logistic and linear regression models

	**Elevated CES-D symptoms**	**Quality of life (CASP-12)**	**Life satisfaction**
Sleeping – Less	3.33*** [2.64,4.21]	-1.90*** [-2.34,-1.45]	-0.80*** [-0.97,-0.62]
Sleeping – Same	*Ref*	*Ref*	*Ref*
Sleeping – More	1.64** [1.16,2.32]	-1.05*** [-1.61,-0.48]	-0.41** [-0.66,-0.16]
Female	1.57*** [1.25,1.97]	-0.39* [-0.72,-0.06]	-0.24*** [-0.37,-0.11]
Age	0.98** [0.97,0.99]	-0.01 [-0.03,0.02]	0.01* [0.00,0.02]
Non-White	0.59 [0.32,1.10]	0.89 [-0.05,1.83]	0.61*** [0.26,0.96]
Medium education (Ref: High)	1.15 [0.87,1.52]	0.21 [-0.16,0.57]	0.26** [0.09,0.43]
Low education	1.04 [0.75,1.43]	0.55* [0.07,1.03]	0.43*** [0.23,0.63]
2nd lowest quintile (Ref: lowest)	0.75 [0.52,1.08]	0.53 [-0.09,1.15]	0.12 [-0.12,0.37]
3rd wealth quintile	0.91 [0.65,1.28]	0.93** [0.31,1.55]	0.06 [-0.18,0.30]
4th wealth quintile	0.80 [0.56,1.14]	0.15 [-0.46,0.77]	-0.16 [-0.40,0.09]
Highest wealth quintile	0.87 [0.60,1.25]	0.54 [-0.07,1.14]	-0.20 [-0.43,0.04]
Employed (Ref: Retired)	0.64** [0.45,0.90]	0.28 [-0.20,0.76]	0.31** [0.11,0.51]
Furloughed	0.56* [0.34,0.91]	-0.05 [-0.69,0.59]	0.10 [-0.20,0.40]
Other employment	1.63* [1.09,2.44]	-1.35*** [-2.11,-0.59]	-0.39* [-0.71,-0.08]
Always with enough food	0.66** [0.50,0.87]	1.65*** [1.15,2.16]	0.30** [0.09,0.52]
Living with partner	0.63*** [0.51,0.78]	-0.05 [-0.41,0.30]	0.15* [0.00,0.31]
Infrequent contacts	1.23 [0.85,1.78]	-0.11 [-0.80,0.59]	0.04 [-0.22,0.31]
Negative experiences of COVID-19	1.20 [0.82,1.77]	-0.22 [-0.83,0.39]	-0.18 [-0.46,0.10]
Clinically vulnerable to COVID-19	1.19 [0.96,1.47]	-0.36* [-0.70,-0.02]	-0.01 [-0.15,0.14]
Disability	1.85*** [1.44,2.39]	-1.11*** [-1.59,-0.63]	-0.02 [-0.21,0.17]
Pre-pandemic elevated depression	4.69*** [3.52,6.26]	0.62*** [0.59,0.66]	0.42*** [0.38,0.45]
Constant		8.30*** [7.04,9.57]	3.70*** [3.26,4.15]
Observations	*4,963*	*4,706*	*4,652*

Sources: ELSA, COVID-19 sub-study Wave 1 (June/July 2020) and Wave 9 (2018/19).
Odds Ratios [and 95% confidence intervals (CIs)] reported for elevated depressive symptoms, and Beta coefficients [and 95% CIs] for the continuous outcome variables 'Quality of life' and 'Life Satisfaction'. For both continous outcomes, the relevant health questions in Wave 9 were asked in the self-completion questionnaire (hence, the smaller sample size). * $p < 0.05$, ** $p < 0.01$, *** $p < 0.001$. Weighted data.

Supplementary Table S5. Cross-sectional associations between changes in drinking and mental health. Fully-adjusted logistic and linear regression models

	**Elevated CES-D symptoms**	**Quality of life (CASP-12)**	**Life satisfaction**
Not drinking alcohol	1.74*** [1.37,2.22]	-0.64** [-1.03,-0.24]	-0.21** [-0.37,-0.06]
Drinking – Less	1.57** [1.12,2.20]	-0.55* [-1.02,-0.08]	-0.29** [-0.48,-0.09]
Drinking – Same	*Ref*	*Ref*	*Ref*
Drinking – More	1.73*** [1.28,2.35]	-0.97*** [-1.43,-0.51]	-0.39*** [-0.58,-0.20]
Female	1.61*** [1.29,2.00]	-0.47** [-0.79,-0.14]	-0.28*** [-0.42,-0.15]
Age	0.97*** [0.96,0.99]	0.00 [-0.02,0.03]	0.02** [0.01,0.03]
Non-White	0.63 [0.35,1.14]	0.86 [-0.08,1.80]	0.57** [0.22,0.93]
Medium education (Ref: High)	1.12 [0.85,1.48]	0.24 [-0.12,0.61]	0.27** [0.10,0.44]
Low education	0.99 [0.72,1.36]	0.61* [0.13,1.09]	0.44*** [0.23,0.65]
2nd lowest quintile (Ref: lowest)	0.77 [0.54,1.11]	0.55 [-0.07,1.17]	0.15 [-0.10,0.40]
3rd wealth quintile	0.93 [0.66,1.29]	0.92** [0.30,1.54]	0.08 [-0.17,0.32]
4th wealth quintile	0.80 [0.57,1.12]	0.17 [-0.44,0.79]	-0.13 [-0.38,0.12]
Highest wealth quintile	0.86 [0.60,1.22]	0.59 [-0.03,1.20]	-0.15 [-0.40,0.09]
Employed (Ref: Retired)	0.64** [0.46,0.88]	0.35 [-0.13,0.83]	0.33** [0.13,0.53]
Furloughed	0.63* [0.40,0.99]	-0.21 [-0.86,0.44]	0.04 [-0.26,0.33]
Other employment	1.64* [1.11,2.43]	-1.38*** [-2.14,-0.62]	-0.42* [-0.74,-0.10]
Always with enough food	0.56*** [0.43,0.73]	1.95*** [1.46,2.45]	0.43*** [0.22,0.65]
Living with partner	0.65*** [0.53,0.81]	-0.09 [-0.45,0.27]	0.14 [-0.01,0.29]
Infrequent contacts	1.25 [0.85,1.82]	-0.13 [-0.86,0.59]	0.03 [-0.25,0.30]
Negative experiences of COVID-19	1.24 [0.84,1.82]	-0.30 [-0.91,0.30]	-0.21 [-0.49,0.08]
Clinically vulnerable to COVID-19	1.23 [0.99,1.52]	-0.41* [-0.75,-0.07]	-0.04 [-0.18,0.11]
Disability	1.92*** [1.50,2.46]	-1.20*** [-1.69,-0.72]	-0.08 [-0.28,0.11]
Pre-pandemic elevated depression	5.07*** [3.83,6.71]	0.64*** [0.60,0.67]	0.43*** [0.39,0.46]
Constant		7.70*** [6.42,8.98]	3.51*** [3.06,3.97]
Observations	*4,959*	*4,705*	*4,651*

Sources: ELSA, COVID-19 sub-study Wave 1 (June/July 2020) and Wave 9 (2018/19).
Odds Ratios [and 95% confidence intervals (CIs)] reported for elevated depressive symptoms, and Beta coefficients [and 95% CIs] for the continuous outcome variables 'Quality of life' and 'Life Satisfaction'. For both continous outcomes, the relevant health questions in Wave 9 were asked in the self-completion questionnaire (hence, the smaller sample size). * p < 0.05, ** p < 0.01, *** p < 0.001. Weighted data

Supplementary Table S6. Longitudinal associations between changes in physical activity and mental health. Fully-adjusted logistic and linear regression models

	**Elevated CES-D symptoms**	**Quality of life (CASP-12)**	**Life satisfaction**
Physical activity – Less	1.38^{**} [1.13,1.68]	-0.71^{***} [-1.07,-0.34]	-0.24^{**} [-0.40,-0.07]
Physical activity – Same	*Ref*	*Ref*	*Ref*
Physical activity – More	0.83 [0.64,1.08]	0.48^{*} [0.07,0.88]	0.11 [-0.07,0.30]
Female	1.42^{***} [1.17,1.72]	-0.69^{***} [-1.01,-0.36]	-0.16^{*} [-0.31,-0.01]
Age	0.97^{***} [0.96,0.98]	0.03^{*} [0.00,0.05]	0.02^{***} [0.01,0.03]
Non-White	0.89 [0.55,1.43]	0.91 [-0.18,2.00]	0.08 [-0.34,0.49]
Medium education (Ref: High)	1.16 [0.91,1.48]	0.01 [-0.37,0.39]	0.03 [-0.14,0.19]
Low education	1.00 [0.75,1.32]	0.77^{**} [0.27,1.27]	0.39^{***} [0.20,0.58]
2nd lowest quintile (Ref: lowest)	0.87 [0.63,1.20]	0.20 [-0.44,0.85]	-0.08 [-0.37,0.21]
3rd wealth quintile	0.92 [0.67,1.25]	0.23 [-0.39,0.84]	-0.17 [-0.42,0.08]
4th wealth quintile	0.87 [0.63,1.19]	0.12 [-0.49,0.74]	-0.15 [-0.42,0.12]
Highest wealth quintile	0.85 [0.60,1.20]	0.44 [-0.20,1.09]	-0.21 [-0.47,0.04]
Employed (Ref: Retired)	0.81 [0.61,1.06]	0.51^{*} [0.06,0.96]	0.17 [-0.03,0.37]
Furloughed	1.50 [0.84,2.70]	-1.88^{**} [-3.09,-0.68]	0.18 [-0.30,0.67]
Other employment	2.07^{***} [1.41,3.04]	-0.23 [-1.02,0.56]	-0.16 [-0.50,0.17]
Always with enough food	0.47^{***} [0.34,0.65]	2.20^{***} [1.54,2.85]	0.89^{***} [0.58,1.20]
Living with partner	0.62^{***} [0.51,0.75]	-0.01 [-0.38,0.35]	0.24^{*} [0.06,0.42]
Infrequent contacts	0.99 [0.73,1.34]	-0.31 [-0.97,0.34]	-0.20 [-0.47,0.07]
Negative experiences of COVID-19	1.03 [0.74,1.42]	-0.17 [-0.84,0.51]	-0.07 [-0.35,0.22]
Clinically vulnerable to COVID-19	1.09 [0.90,1.31]	-0.15 [-0.50,0.20]	0.06 [-0.10,0.21]
Disability	1.86^{***} [1.49,2.32]	-1.18^{***} [-1.66,-0.71]	-0.33^{**} [-0.54,-0.13]
Pre-pandemic elevated depression	5.02^{***} [3.87,6.52]	0.67^{***} [0.64,0.71]	0.41^{***} [0.37,0.46]
Constant		5.84^{***} [4.63,7.04]	3.06^{***} [2.60,3.53]
Observations	*4,959*	*4,704*	*4,653*

Sources: ELSA, COVID-19 sub-study Wave 1 (June/July 2020), COVID-19 sub-study Wave 2 (November/December 2020) and Wave 9 (2018/19).

Odds Ratios [and 95% confidence intervals (CIs)] reported for elevated depressive symptoms, and Beta coefficients [and 95% CIs] for the continuous outcome variables 'Quality of life' and 'Life Satisfaction'. For both continous outcomes, the relevant health questions in Wave 9 were asked in the self-completion questionnaire (hence, the smaller sample size). * $p < 0.05$, ** $p < 0.01$, *** $p < 0.001$. Weighted data

Supplementary Table S7. Longitudinal associations between changes in eating and mental health. Fully-adjusted logistic and linear regression models

	**Elevated CES-D symptoms**	**Quality of life (CASP-12)**	**Life satisfaction**
Eating – Less	1.34* [1.02,1.77]	-0.12 [-0.75,0.51]	-0.07 [-0.32,0.19]
Eating – Same	*Ref*	*Ref*	*Ref*
Eating – More	1.45** [1.15,1.84]	-0.72** [-1.17,-0.26]	-0.26** [-0.46,-0.06]
Female	1.40*** [1.16,1.70]	-0.67*** [-1.01,-0.34]	-0.14 [-0.29,0.01]
Age	0.98*** [0.96,0.99]	0.02 [-0.00,0.04]	0.02** [0.01,0.03]
Non-White	0.96 [0.59,1.54]	0.93 [-0.14,2.00]	0.06 [-0.35,0.46]
Medium education (Ref: High)	1.17 [0.92,1.49]	-0.01 [-0.39,0.38]	0.02 [-0.14,0.19]
Low education	1.01 [0.76,1.33]	0.76** [0.25,1.27]	0.39*** [0.19,0.58]
2nd lowest quintile (Ref: lowest)	0.88 [0.64,1.21]	0.19 [-0.45,0.83]	-0.08 [-0.37,0.21]
3rd wealth quintile	0.91 [0.67,1.24]	0.23 [-0.38,0.85]	-0.17 [-0.42,0.08]
4th wealth quintile	0.86 [0.63,1.18]	0.13 [-0.49,0.75]	-0.14 [-0.41,0.13]
Highest wealth quintile	0.84 [0.60,1.19]	0.47 [-0.18,1.11]	-0.20 [-0.46,0.05]
Employed (Ref: Retired)	0.81 [0.61,1.06]	0.50* [0.05,0.95]	0.17 [-0.03,0.37]
Furloughed	1.49 [0.85,2.62]	-1.86** [-3.08,-0.64]	0.19 [-0.29,0.67]
Other employment	2.08*** [1.42,3.06]	-0.26 [-1.07,0.54]	-0.19 [-0.52,0.14]
Always with enough food	0.48*** [0.34,0.66]	2.21*** [1.55,2.87]	0.89*** [0.58,1.20]
Living with partner	0.62*** [0.51,0.76]	-0.04 [-0.40,0.33]	0.23* [0.04,0.41]
Infrequent contacts	1.03 [0.75,1.40]	-0.41 [-1.07,0.25]	-0.21 [-0.48,0.06]
Negative experiences of COVID-19	1.01 [0.73,1.40]	-0.18 [-0.87,0.51]	-0.07 [-0.36,0.23]
Clinically vulnerable to COVID-19	1.10 [0.92,1.33]	-0.20 [-0.55,0.16]	0.05 [-0.10,0.20]
Disability	1.91*** [1.54,2.38]	-1.21*** [-1.69,-0.73]	-0.35*** [-0.55,-0.14]
Pre-pandemic elevated depression	4.86*** [3.74,6.31]	0.68*** [0.64,0.71]	0.41*** [0.37,0.46]
Constant		5.75*** [4.55,6.95]	3.05*** [2.59,3.51]
Observations	*4,966*	*4,710*	*4,659*

Sources: ELSA, COVID-19 sub-study Wave 1 (June/July 2020), COVID-19 sub-study Wave 2 (November/December 2020) and Wave 9 (2018/19).

Odds Ratios [and 95% confidence intervals (CIs)] reported for elevated depressive symptoms, and Beta coefficients [and 95% CIs] for the continuous outcome variables 'Quality of life' and 'Life Satisfaction'. For both continous outcomes, the relevant health questions in Wave 9 were asked in the self-completion questionnaire (hence, the smaller sample size). * $p < 0.05$, ** $p < 0.01$, *** $p < 0.001$. Weighted data

Supplementary Table S8. Longitudinal associations between changes in sleeping and mental health. Fully-adjusted logistic and linear regression models

	**Elevated CES-D symptoms**	**Quality of life (CASP-12)**	**Life satisfaction**
Sleeping – Less	2.42^{***} [1.95,3.00]	-1.40^{***} [-1.84,-0.97]	-0.47^{***} [-0.66,-0.29]
Sleeping – Same	*Ref*	*Ref*	*Ref*
Sleeping – More	1.46^{*} [1.08,1.98]	-0.89^{**} [-1.51,-0.27]	-0.32^{*} [-0.59,-0.06]
Female	1.39^{***} [1.14,1.69]	-0.65^{***} [-0.98,-0.32]	-0.14 [-0.29,0.02]
Age	0.98^{***} [0.97,0.99]	0.01 [-0.01,0.04]	0.02^{**} [0.01,0.03]
Non-White	0.91 [0.55,1.51]	1.01 [-0.09,2.10]	0.09 [-0.32,0.50]
Medium education (Ref: High)	1.20 [0.94,1.54]	-0.05 [-0.43,0.34]	0.01 [-0.15,0.18]
Low education	1.00 [1.00,1.00]	0.70^{**} [0.19,1.21]	0.37^{***} [0.18,0.57]
2nd lowest quintile (Ref: lowest)	0.88 [0.63,1.22]	0.19 [-0.45,0.83]	-0.09 [-0.38,0.20]
3rd wealth quintile	0.92 [0.67,1.25]	0.26 [-0.35,0.87]	-0.17 [-0.42,0.08]
4th wealth quintile	0.90 [0.65,1.25]	0.10 [-0.52,0.72]	-0.17 [-0.43,0.10]
Highest wealth quintile	0.89 [0.62,1.26]	0.44 [-0.20,1.08]	-0.22 [-0.48,0.03]
Employed (Ref: Retired)	0.81 [0.61,1.07]	0.48^{*} [0.03,0.92]	0.16 [-0.04,0.36]
Furloughed	1.41 [0.78,2.56]	-1.79^{**} [-3.02,-0.56]	0.21 [-0.25,0.68]
Other employment	2.10^{***} [1.44,3.06]	-0.24 [-1.04,0.55]	-0.18 [-0.51,0.16]
Always with enough food	0.50^{***} [0.36,0.70]	2.08^{***} [1.42,2.74]	0.85^{***} [0.53,1.16]
Living with partner	0.62^{***} [0.51,0.75]	-0.03 [-0.39,0.33]	0.23^{*} [0.05,0.41]
Infrequent contacts	1.06 [0.77,1.45]	-0.45 [-1.11,0.21]	-0.22 [-0.49,0.05]
Negative experiences of COVID-19	0.98 [0.71,1.35]	-0.13 [-0.81,0.55]	-0.05 [-0.35,0.24]
Clinically vulnerable to COVID-19	1.08 [0.90,1.31]	-0.17 [-0.52,0.18]	0.06 [-0.09,0.22]
Disability	1.82^{***} [1.45,2.27]	-1.12^{***} [-1.60,-0.65]	-0.31^{**} [-0.51,-0.11]
Pre-pandemic elevated depression	4.56^{***} [3.49,5.96]	0.66^{***} [0.63,0.70]	0.41^{***} [0.36,0.45]
Constant		6.43^{***} [5.20,7.66]	3.23^{***} [2.76,3.70]
Observations	*4,965*	*4,709*	*4,658*

Sources: ELSA, COVID-19 sub-study Wave 1 (June/July 2020), COVID-19 sub-study Wave 2 (November/December 2020) and Wave 9 (2018/19).

Odds Ratios [and 95% confidence intervals (CIs)] reported for elevated depressive symptoms, and Beta coefficients [and 95% CIs] for the continuous outcome variables ‘Quality of life’ and ‘Life Satisfaction’. For both continous outcomes, the relevant health questions in Wave 9 were asked in the self-completion questionnaire (hence, the smaller sample size). * $p < 0.05$, ** $p < 0.01$, *** $p < 0.001$. Weighted data

Supplementary Table S9. Longitudinal associations between changes in drinking and mental health. Fully-adjusted logistic and linear regression models

	Elevated CES-D symptoms	Quality of life (CASP-12)	Life satisfaction
Not drinking alcohol	1.15 [0.93,1.43]	-0.02 [-0.41,0.37]	0.03 [-0.16,0.21]
Drinking – Less	1.17 [0.89,1.54]	-0.22 [-0.68,0.25]	-0.20 [-0.42,0.01]
Drinking – Same	*Ref*	*Ref*	*Ref*
Drinking – More	1.40* [1.04,1.88]	-0.54* [-1.06,-0.02]	-0.23* [-0.46,-0.00]
Female	1.45*** [1.19,1.76]	-0.75*** [-1.09,-0.42]	-0.19* [-0.34,-0.03]
Age	0.97*** [0.96,0.99]	0.02 [-0.00,0.04]	0.02** [0.01,0.03]
Non-White	0.95 [0.59,1.53]	0.88 [-0.20,1.96]	0.02 [-0.39,0.43]
Medium education (Ref: High)	1.18 [0.93,1.50]	-0.03 [-0.42,0.35]	0.01 [-0.16,0.18]
Low education	1.02 [0.77,1.35]	0.72** [0.21,1.22]	0.36*** [0.17,0.56]
2nd lowest quintile (Ref: lowest)	0.87 [0.64,1.20]	0.21 [-0.44,0.85]	-0.08 [-0.37,0.21]
3rd wealth quintile	0.90 [0.66,1.22]	0.28 [-0.34,0.89]	-0.15 [-0.40,0.11]
4th wealth quintile	0.87 [0.63,1.19]	0.14 [-0.48,0.76]	-0.13 [-0.40,0.13]
Highest wealth quintile	0.83 [0.60,1.17]	0.51 [-0.14,1.16]	-0.18 [-0.43,0.08]
Employed (Ref: Retired)	0.81 [0.62,1.06]	0.49* [0.04,0.94]	0.17 [-0.03,0.37]
Furloughed	1.49 [0.85,2.62]	-1.87** [-3.09,-0.65]	0.19 [-0.29,0.66]
Other employment	2.06*** [1.41,3.01]	-0.27 [-1.07,0.54]	-0.20 [-0.54,0.13]
Always with enough food	0.46*** [0.34,0.64]	2.24*** [1.59,2.90]	0.91*** [0.59,1.22]
Living with partner	0.62*** [0.51,0.76]	-0.02 [-0.39,0.34]	0.23* [0.05,0.41]
Infrequent contacts	1.03 [0.76,1.39]	-0.41 [-1.06,0.25]	-0.21 [-0.48,0.06]
Negative experiences of COVID-19	1.02 [0.74,1.41]	-0.18 [-0.86,0.50]	-0.05 [-0.35,0.24]
Clinically vulnerable to COVID-19	1.12 [0.93,1.36]	-0.23 [-0.59,0.12]	0.03 [-0.13,0.18]
Disability	1.93*** [1.55,2.40]	-1.22*** [-1.70,-0.74]	-0.36*** [-0.56,-0.15]
Pre-pandemic elevated depression	4.94*** [3.81,6.40]	0.68*** [0.64,0.71]	0.41*** [0.37,0.46]
Constant		5.71*** [4.48,6.95]	3.06*** [2.57,3.55]
Observations	*4,965*	*4,709*	*4,658*

Sources: ELSA, COVID-19 sub-study Wave 1 (June/July 2020), COVID-19 sub-study Wave 2 (November/December 2020) and Wave 9 (2018/19).

Odds Ratios [and 95% confidence intervals (CIs)] reported for elevated depressive symptoms, and Beta coefficients [and 95% CIs] for the continuous outcome variables ‘Quality of life’ and ‘Life Satisfaction’. For both continous outcomes, the relevant health questions in Wave 9 were asked in the self-completion questionnaire (hence, the smaller sample size). * $p < 0.05$, ** $p < 0.01$, *** $p < 0.001$. Weighted data
